# Oxidative Stress, DNA Damage, DNA Repair Inhibition, and Apoptosis Induced by Lead and Cadmium Combined Exposure in TK6 Cells

**DOI:** 10.3390/toxics14040341

**Published:** 2026-04-18

**Authors:** Xin Liu, Zhiyuan Han, Kuibin Han, Yuhan Pang, Xiaoyue Zhao, Yuting Wang, Xiaoyan Wu, Tuanwei Wang

**Affiliations:** Department of Occupational and Environmental Health, School of Public Health, Shandong Second Medical University, Weifang 261053, China

**Keywords:** lead exposure, cadmium exposure, combined exposure, oxidative stress, DNA damage, DNA repair, apoptosis

## Abstract

Lead (Pb) and cadmium (Cd) are common environmental pollutants. Our previous population study revealed a significant positive association between Pb and Cd exposure and the micronuclei frequency among lead smelting workers. However, the underlying mechanisms remain unclear. In this study, human lymphoblastoid TK6 cells were used to investigate the genotoxicity and its mechanisms induced by individual or combined exposure to Pb and Cd. Our results showed that Pb and Cd exposure, alone or in combination, triggered oxidative stress, as evidenced by reduced antioxidant enzyme activity (GSH, SOD and CAT) and increased content of ROS and GSSG. Both metals induced pronounced DNA damage, as shown by elevated Tail DNA% in the Comet assay and γ-H2AX fluorescence intensity. Furthermore, Pb and/or Cd exposure caused inhibition of the DNA repair proteins, including BRCA1, CtIP, RAD52, and XRCC2, indicating impaired DNA repair capacity; and upregulated Bax expression and the Bax/Bcl-2 ratio and Caspase-3 with downregulation of Bcl-2. Notably, Pb and Cd co-exposure produced an antagonistic effect, modulating oxidative stress indicators, cell-cycle arrest, DNA damage markers, DNA repair and apoptosis-related proteins. These findings demonstrate that Pb and Cd induce oxidative stress, DNA damage, inhibition of DNA repair, and apoptosis in TK6 cells. Our study provides new insights into the mechanisms of heavy metal combined exposure–induced genotoxicity and identifies potential molecular targets for intervention.

## 1. Introduction

Lead (Pb) and cadmium (Cd) are among the most pervasive environmental pollutants due to their extensive use in mining, smelting, battery manufacturing, and electronic waste recycling. These two metals accumulate persistently in soil, water, and the food chain, resulting in chronic human exposure and long-term health risks [1,2]. The International Agency for Research on Cancer (IARC) classifies Cd as a human carcinogen (G1) and Pb as a probable human carcinogen (G2A), highlighting their significant toxicity and public health concerns [3,4].

Growing evidence indicates that both metals exert strong genotoxic effects. Pb has been shown to impair DNA synthesis, interfere with chromosomal segregation, and induce DNA strand breaks, thereby compromising genomic stability [5,6]. Cd similarly causes DNA single- and double-strand breaks, chromosomal aberrations, and alterations in gene expression [7,8]. Epidemiological studies and animal experiments demonstrate elevated oxidative DNA damage and micronuclei formation in populations or animals exposed to Pb or Cd, underscoring their contribution to genomic stability and disease risk [9].

Oxidative stress is considered a major trigger of Pb- and Cd-induced cytotoxicity. Both metals promote the generation of reactive oxygen species (ROS), disrupt the balance of the oxidation-antioxidant system, and deplete molecules such as superoxide dismutase (SOD) and glutathione (GSH). Previous studies on occupationally exposed workers have shown that Pb toxicity is mainly mediated through oxidative stress and nitrative stress, leading to extensive biomolecular damage. Elevated blood lead levels (BLLs) are associated with increased lipid peroxidation, depletion of thiol groups, accumulation of protein carbonyls, and DNA strand breaks, reflecting multifactorial cellular damage [10]. In lymphocytes, Pb exposure exacerbates DNA instability, and the presence of Pb has also been shown to affect the action of certain antioxidants on pro-oxidants, further aggravating genotoxicity [11]. Overall, these findings emphasize that the mechanisms of Pb toxicity are primarily driven by oxidative imbalance, biomolecular degradation, and genotoxic stress. Excessive ROS leads to lipid peroxidation, protein oxidation, and oxidative DNA damage, including 8-oxo-dG formation [12]. In addition, Cd has been shown to suppress antioxidant-related gene expression and enhance Pb-induced oxidative damage in a dose- and time-dependent manner [13]. These findings suggest that oxidative stress may play a central role in mediating the genotoxic effects of Pb and Cd exposure.

Apoptosis is another key outcome of heavy metal toxicity. Cd induces apoptosis in various cell types [14,15,16,17], and may exert transgenerational effects via epigenetic modifications of apoptosis-related genes and microRNAs [18]. Pb exposure also triggers apoptosis through ROS accumulation, mitochondrial dysfunction, and altered expression of Bax, Bcl-2, and Caspases [19]. Although some studies suggest that Pb and Cd co-exposure may exacerbate the progression of apoptosis [20,21,22,23], there is a lack of systematic study on the regulatory mechanisms linking oxidative stress, DNA damage, the DNA repair process and the apoptosis pathway under co-exposure to these two metals. A large number of studies have confirmed that Pb and Cd can significantly induce DNA base lesions, gene mutations, and oxidative damage, and downregulate the transcription levels of key repair genes such as XRCC1, ERCC1, and hOGG1 [24,25]. However, the synergistic mechanism of these on DNA damage response (DDR) and repair network inhibition has not yet been elucidated.

In this study, the human lymphoblastoid cell line TK6 was selected as the test model. This cell line is derived from human peripheral blood lymphocytes and possesses high differentiation potential and genetic stability. Compared with other cell models, TK6 retains wild-type p53 function, with complete cell cycle regulation and DNA damage response mechanisms, allowing for a more accurate simulation of the biological effects of exogenous toxins on human cells. It provides a reliable experimental model for assessing the genotoxicity of lead and cadmium co-exposure [26,27]. By detecting oxidative stress indicators, DNA damage markers, DNA repair and apoptosis proteins, this study aims to elucidate the molecular basis of heavy metal combined exposure–induced genotoxicity. These findings may provide new insights into the toxicity of Pb and Cd co-exposure and identify potential molecular targets for prevention.

## 2. Materials and Methods

### 2.1. Cell Culture and Treatment

TK6 cells were purchased from Procell Life Technology Co., Ltd. (Procell, Wuhan, China, CL-0813), they are suspension cells and lymphoid like cells, and have high differentiation potential and genomic stability, they are derived from human peripheral blood lymphocytes, and cultured in RPMI 1640 medium (Solarbio, Beijing, China, 31800) supplemented with 10% fetal bovine serum and 1% penicillin-streptomycin (100 U/mL penicillin and 100 μg/mL streptomycin, Solarbio, Beijing, China, P1400) at 37 °C and 5% CO_2_, with the culture medium replaced every two days. In this study, after determining the toxic concentrations of Pb and Cd based on their half lethal concentration (IC_50_), the TK6 cells were divided into five groups: the control group, Pb group (480 μM lead acetate), Cd group (33 μM cadmium chloride), mixed group (480 μM lead acetate + 33 μM cadmium chloride), and resveratrol group (mixed group + 10 μM resveratrol). Cells at a density of 1.0 × 10^4^ or 1.0 × 10^6^ cells/mL were evenly seeded into 96-well plates or 6-well plates (NEST, Wuxi, China, 701011 and 801004), and all treatments were conducted for 24 h.

### 2.2. CCK-8 Assay

Cell viability was assessed using the CCK-8 assay (Beyotime, Shanghai, China; C0042). Briefly, 100 μL of a cell suspension (1.0 × 10^5^ cells/mL) was seeded into each well of a 96-well plate and then applied to evaluate dose-dependent manners on cell survival, apoptosis and enzyme activities, with lead acetate [Pb(CH_3_COO)_2_] (Aladdin, Shanghai, China, L118642) ranging from 0 to 1600 μM (0, 100, 200, 400, 800, 1600 μM) and cadmium chloride (CdCl_2_) (Macklin, Shanghai, China, C805625) from 0 to 160 μM (0, 10, 20, 40, 80, 160 μM). After 24 h of exposure, the culture medium was removed and replaced with fresh medium containing 10 μL of CCK-8 reagent per well, followed by incubation for an additional 2 h. The absorbance values at 450 nm were measured using a microplate reader (Molecular Devices, Shanghai, China), and cell viability was calculated accordingly.

### 2.3. Intracellular ROS Detection

Intracellular ROS levels were detected using the fluorescent probe DCFH-DA. TK6 cells from the different treatment groups were washed with cold PBS (Solarbio, Beijing, China, P1020) buffer, and according to the instructions of the ROS detection kit (Beyotime, Shanghai, China; S0033S), the cells were incubated in the dark at 37 °C for 40 min using DCFH-DA (10 μM). The cells underwent three PBS washes and subsequent DAPI staining for 5 min. Fluorescence intensity was measured under a fluorescence microscope (Leica, Wetzlar, Germany) with an excitation wavelength of 488 nm and an emission wavelength of 525 nm. ImageJ 150i software was used for data analysis.

### 2.4. Oxidative Stress Indicators

This study evaluated oxidative damage by measuring the content of malondialdehyde (MDA), oxidized and reduced glutathione (GSSG/GSH), and the activity of antioxidant enzymes superoxide dismutase (SOD) and catalase (CAT) using commercial kits (Beyotime, Shanghai, China). TK6 cells from the different treatment groups were collected and washed with ice-precooled PBS, followed by lysis in ice-cold RIPA buffer for 30 min. Subsequent centrifugation of the lysates at 12,000 rpm for 10 min yielded supernatants for determining MDA (μmol/mg protein), GSSG and GSH content (μmol/mg protein), SOD (units/mg protein), and CAT activity (units/mg). Specifically, when determining MDA, MDA detection working solution and TBA storage solution were firstly prepared, then an appropriate amount of MDA standard were diluted with ultrapure water to prepare a series of standard working solutions with final concentrations of 1, 2, 5, 10, 20, and 50 μM, respectively; 0.1 mL of lysis buffer (blank control), 0.1 mL of series concentration standard (standard curve), or 0.1 mL of test sample were added into the centrifuge tube respectively; and then 0.2 mL of MDA detection working solution was added in the centrifuge tube, mixed well, heated at 100 °C for 15 min, cooled the water bath to room temperature, and centrifuged 1000× *g* at room temperature for 10 min. 200 μL of supernatant was transferred to a 96-well plate, and the absorbance was measured at 532 nm (A532) with a microplate reader. For measuring GSSG and GSH, briefly, samples or standards were added to the 96-well plate in turn, and mixed well. 150 μL of total glutathione detection solution was added, mixed well, and incubated at room temperature for 5 min. Then, 50 μL of 0.5 mg/mL NADPH solution was added and mixed well; the absorbance was measured at 412 nm immediately. When determining SOD, 20 μL of sample supernatant was added into a 96-well plate, 20 μL and 40 μL of SOD detection buffer was added to blank control 1 and blank control 2, then 160 μL of enzyme working solution was added to all samples, and finally 20 μL of reaction initiation working solution was added, and incubated the mixture at 37 °C for 30 min, then measured the absorbance at 450 nm. For measuring CAT, a 250 mM hydrogen peroxide solution was prepared, the A240 was determined, and the standard curve was established. The sample, catalase detection buffer and 250 mM hydrogen peroxide solution were added in turn, mixed and reacted at 25 °C for 5 min. 450 μL catalase reaction stop solution was added and mixed upside down to terminate the reaction. A total of 40 μL catalase detection buffer was added, and then 10 μL of the above reaction system was added, which had been terminated and mixed, and mixed well. 10 μL from the 50 μL system from the previous step was added into one well in a 96-well plate, and then 200 μL of the working solution was added. A520 was measured after 15 min of incubation at 25 °C. Protein concentrations were determined using a BCA protein assay kit (Beyotime, Shanghai, China; P0011).

### 2.5. γ-H2AX Levels

DNA damage was further assessed using a γ-H2AX detection kit (Beyotime, Shanghai, China; C2035S), which quantifies γ-H2AX levels, a well-established biomarker for DNA double-strand breaks. TK6 cells from the different treatment groups were collected by centrifugation, fixed on glass slides, and washed with PBS. The slides were then blocked with immunostaining blocking buffer at room temperature for 20 min. Subsequently, an appropriate amount of γ-H2AX rabbit monoclonal antibody (1:100) was added dropwise to the slides, followed by incubation at 4 °C overnight. After washing three times with washing buffer, a fluorescently conjugated secondary antibody (1:1000, Beyotime) was added dropwise and incubated for 1 h at room temperature. Nuclei were stained with DAPI and visualized under a fluorescence microscope (Leica, Wetzlar, Germany). γ-H2AX fluorescence intensity was analyzed using ImageJ 150i software, (https://imagej.net/ij/, accessed on 8 April 2026).

### 2.6. Alkaline Comet Assay

DNA damage was further evaluated using a Comet assay kit (Beyotime, Shanghai, China; C2041M). First, a base gel layer was prepared by spreading 30 μL of 1% agarose gel (pre-warmed to 45 °C) onto the slide and allowing it to solidify at 4 °C for 10 min. TK6 cells from the different treatment groups were collected, washed, and resuspended in PBS. A 10 μL aliquot of cell suspension was mixed with 75 μL of 0.7% low-melting point agarose in a 37 °C water bath, and 70 μL of the mixture was pipetted onto the first layer. The gel was allowed to solidify at 4 °C for 10 min. The slides were then immersed in fresh lysis buffer and incubated at 4 °C for at least 2 h. After rinsing with PBS for 3 min, the slides were placed in electrophoresis buffer for 40 min to allow DNA unwinding. Electrophoresis was subsequently performed at 4 °C for 20 min (25 V and 300 mA). The slides were then transferred to neutralization buffer and neutralized three times at 4 °C, followed by staining with propidium iodide (PI) in the dark for 20 min. Comets were examined under a fluorescence microscope (Leica, Wetzlar, Germany). For each slide, 100 cells were randomly selected, and Tail length and Tail DNA percentage (Tail DNA%) were analyzed using CASP 1.2.3b2 software.

### 2.7. Flow Cytometry

For cell cycle analysis, TK6 cells from the different treatment groups were collected by centrifugation, washed with PBS, fixed with 70% ethanol prechilled on ice, and incubated at 4 °C for at least 2 h. After an additional PBS washing, the cells were stained with propidium iodide (PI) using a cell cycle analysis kit (Beyotime, Shanghai, China; C1052), according to the manufacturer’s instructions. Red fluorescence was acquired on a flow cytometer (C6 plus, BD, Franklin Lakes, NJ, USA) with excitation at 488 nm.

For apoptosis analysis, TK6 cells from the different treatment groups were harvested according to the operating procedures of the Annexin V-FITC apoptosis detection kit (Beyotime, Shanghai, China; C1062L). The cells were harvested and gently resuspended in Annexin V binding buffer. The cell suspension was then stained with Annexin V-FITC and PI. Single-stained controls (Annexin V-FITC only or PI only) were prepared for compensation. Apoptosis was analyzed by flow cytometry (C6 plus, BD, Franklin Lakes, NJ, USA), where Annexin V-FITC was detected as green fluorescence and PI as red fluorescence.

### 2.8. Real-Time Quantitative PCR (RT-qPCR)

The mRNA expression levels of *BRCA1*, *CtIP*, *RAD52*, *XRCC2*, *Bax*, *Bcl-2*, and *Caspase-3* genes in TK6 cells were measured by RT-qPCR. Total RNA was extracted using the Pure Cell Total RNA Isolation Kit V2 (Vazyme, Nanjing, China; RC112), and RNA purity was assessed by measuring the absorbance value at 230, 260, and 280 nm using an ultramicro spectrophotometer (Denovix, Wilmington, DE, USA; DS-11+), and required an A260/A280 ratio between 1.8 and 2.0, and an A260/A230 ratio greater than 2. First-strand cDNA was synthesized from HiScript IV RT SuperMix for qPCR (+gDNA wiper) (Vazyme, Nanjing, China; R423) according to the manufacturer’s instructions. RT-qPCR was performed in a 20 μL PCR reaction mixture containing 10 μLof T ChamQ Universal SYBR qPCR Master Mix (Vazyme, Nanjing, China; Q711), 2 μL of cDNA, 0.4 μLof forward and reverse primers each (final concentration 10 μM), and 7.2 μL of ddH_2_O.

The thermal cycling conditions were as follows: initial denaturation at 95 °C for 5 s, followed by 40 cycles of 95 °C for 10 s and 60 °C for 30 s on a QuantStudio 5 Real-Time PCR system (Applied Biosystems, Carlsbad, CA, USA), with a default melting-curve analysis at the end of amplification. The primer sequences (Sangon Biotech, Shanghai, China) are listed in Table 1. *GAPDH* was used as an internal control. Relative mRNA expression levels were calculated using the 2^−ΔΔCt^ method.

### 2.9. Western Blotting

TK cells were collected, washed twice with PBS from different treatment groups, and lysed on ice for 30 min in RIPA buffer containing PMSF (Beyotime, Shanghai, China; P0013J). Protein concentrations were determined using a BCA Protein Assay Kit (Beyotime, Shanghai, China; P0011). Equal amounts of lysed protein were separated by SDS-polyacrylamide gel electrophoresis (SDS-PAGE) (Affinibody, Wuhan, China, NGN08) and transferred onto PVDF membranes (Beyotime, Shanghai, China; FFP106). The membranes were blocked with rapid blocking solution (Epizyme Biotech, Shanghai, China) for 1 h at room temperature and then incubated overnight at 4 °C with rabbit polyclonal primary antibodies against BRCA1, CtIP, RAD52, XRCC2, Bax, Bcl-2, and Caspase-3 (Affinity, Cincinnati, Ohio, the U.S; all at 1:1000 dilution). After washing three times with TBST for 10 min each time, the membranes were incubated for 45 min at room temperature with HRP-conjugated secondary antibodies (Beyotime, Shanghai, China, 1:2000). Protein bands were visualized using an enhanced chemiluminescence reagent (Biosharp ECL kit, Beijing, China) and quantified by densitometry using ImageJ 150i software.

### 2.10. Statistical Analysis

Statistical analyses were performed using SPSS 26.0. Data are expressed as mean ± standard deviation (SD). Between-group differences were analyzed by one-way analysis of variance (ANOVA), followed by Bonferroni post hoc tests for pairwise comparisons. Based on the *F*- and *p*-values of the interaction term from two-way ANOVA, the type of interaction between factor A and factor B was determined by comparing the combined effect (E_A_ × E_B_) with the sum of the individual effects (E_A_ + E_B_). The criteria were as follows:: synergistic effect, *F* > 5, *p* < 0.05, and E_A_ × E_B_ > E_A_ + E_B_; additive effect, *F* < 5, *p >* 0.05, and E_A_ × E_B_ > E_A_ + E_B_; antagonistic effect, *F* > 5, *p* < 0.05, and E_A_ × E_B_ < E_A_ + E_B_. In addition, to further visualize the interaction between Pb and Cd, interaction plots (profile plots) were generated as described previously [28]. Non-parallel lines (i.e., that diverge from each other) were interpreted as indicating synergistic interaction, whereas approximately parallel lines indicated an additive effect. All experiments were repeated at least in triplicate (*n* ≥ 3), and *p*-values < 0.05 were considered statistically significant.

## 3. Results

### 3.1. Pb and Cd Caused Cell Viability Decrease in TK6 Cells

**Figure 1:**
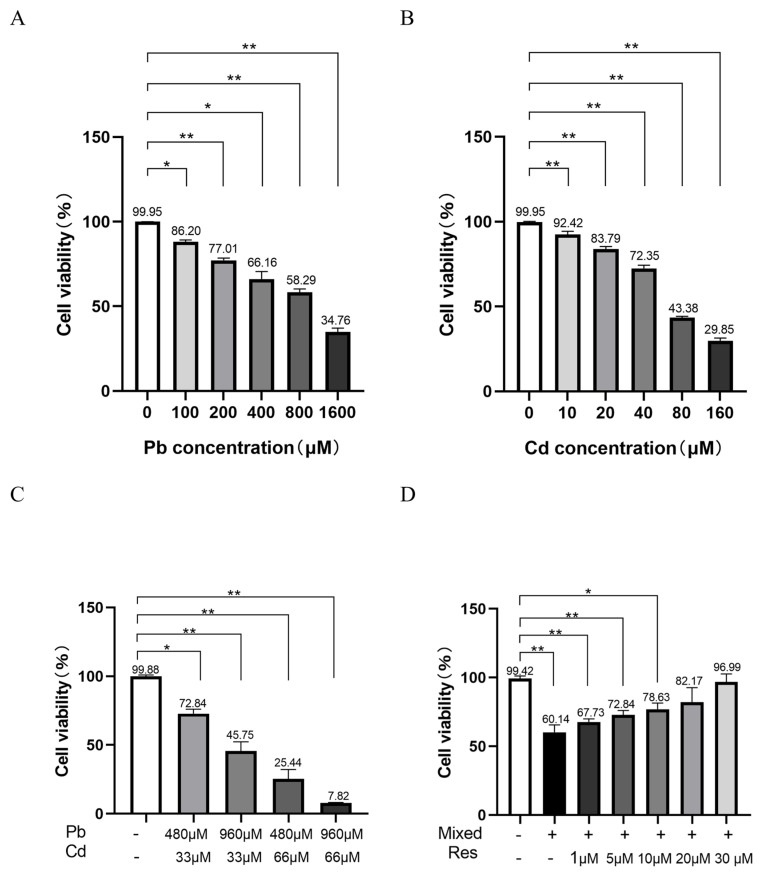
Cytotoxicity of Pb and/or Cd, and the intervention dose of resveratrol in TK6 cells. (**A**). Cell viability of TK6 cells exposed to a series of concentrations of Pb acetate for 24 h. (**B**). Cell viability of TK6 cells exposed to a series of concentrations of Cd chloride for 24 h. (**C**). Cell viability of TK6 cells exposed to a series of concentrations of Pb acetate and Cd chloride for 24 h. (**D**). Cell viability of TK6 cells co-exposed to a series of concentrations of resveratrol and the mixed treatment of Pb acetate and Cd chloride for 24 h. Data were expressed as the mean ± SD (*n* = 3). * *p* < 0.05, ** *p* < 0.01 for Pb and/or Cd groups vs. the control group.

TK6 cells were exposed to a series of concentrations of Pb acetate and Cd chloride (Figure 1A,B). Compared with the control group, cell viability began to decrease at 100 μM Pb and 10 μM Cd, and the survival rate of TK6 cells decreased in a dose-dependent manner with increasing concentration of either metal (Figure 1). Based on the resulting dose–response curves, using IC50 as the criterion, the effects of Pb and Cd co-exposure on cell viability were investigated. Ultimately, 0.5 × IC_50_ values for Pb and Cd (480 μM lead acetate and/or 33 μM cadmium chloride) were selected as the exposure concentration for subsequent experiments, consistent with the previous study [29]. When TK6 cells were co-exposed to series concentrations of resveratrol and the mixed treatment of Pb acetate and Cd chloride, it was found that resveratrol in the mixed group showed a statistically significant difference at 10 μM (*p* < 0.05), it was a critical value, and resveratrol can significantly alleviate the decrease in cell viability caused by lead and cadmium co-exposure (78.63 vs. 60.14%, *p* < 0.05), in addition, there was no significant difference between 10 μM and 20 μM resveratrol (78.63 vs. 82.17%, *p >* 0.05). Furthermore, one previous study indicated that 1 μM resveratrol can significantly alleviate DNA damage in TK6 cells [30]; therefore, 10 μM resveratrol should have beneficial therapeutic effects and was selected as the experimental dose for subsequent studies. The cells were divided into the following groups: the control group, Pb group (480 μM lead acetate), Cd group (33 μM cadmium chloride), the mixed group (480 μM lead acetate + 33 μM cadmium chloride), and the resveratrol group (the mixed group + 10 μM resveratrol).

### 3.2. Pb and Cd Induced Oxidative Stress in TK6 Cells

**Figure 2:**
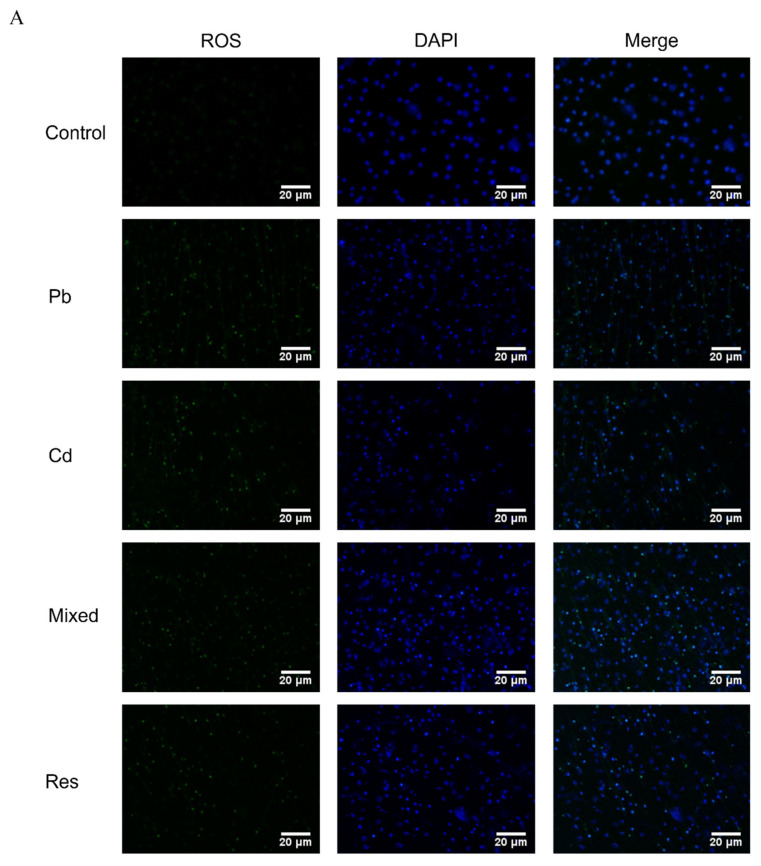

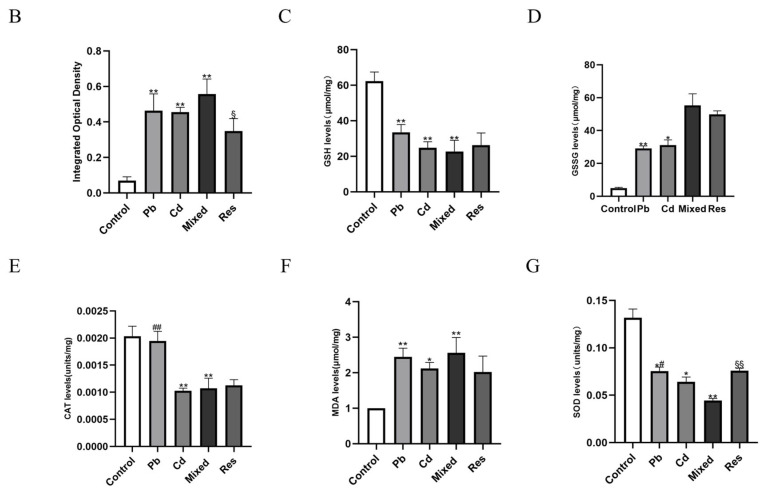
Intracellular ROS levels and oxidative and antioxidative indicators induced by Pb and/or Cd in TK6 cells. (**A**). Representative fluorescence images of intracellular ROS in TK6 cells; (**B**). Quantitative analysis of ROS fluorescence intensity; (**C**–**G**). Intracellular levels of reduced GSH, CAT, SOD, and increased GSSG, MDA respectively. Note: * *p* < 0.05, ** *p* < 0.01 for Pb and/or Cd group vs. the control group; while ^#^ *p* < 0.05, ^##^ *p* < 0.01 for single exposure groups vs. the mixed group; ^§^ *p* < 0.05, ^§§^ *p* < 0.01 for the resveratrol group vs. the mixed group.

The fluorescence probe assay showed that co-exposure to Pb and Cd significantly increased intracellular ROS levels compared with the control group (*p* < 0.05), whereas ROS accumulation was markedly reduced in the Pb-only, Cd-only and resveratrol intervention group relative to the mixed group (*p* < 0.05; Figure 2A,B). Compared with the control group, the Pb or Cd individual group exhibited significantly decreased intracellular GSH content and SOD and CAT activities, accompanied by significant increases in GSSG and MDA levels (*p* < 0.05; Figure 2C–G). In contrast, relative to the mixed group, both the single-metal group and the resveratrol group significantly restored GSH content and SOD activity and reduced GSSG levels (*p* < 0.05; Figure 2C,D,G). Two-way ANOVA further revealed an antagonistic interaction between Pb and Cd on intracellular GSH content and SOD activity (*F*_GSH = 7.373, *p* = 0.026; *F*_SOD = 10.098, *p* = 0.008; Figure S1).

### 3.3. Pb and Cd Aggravated DNA Damage in TK6 Cells

**Figure 3:**
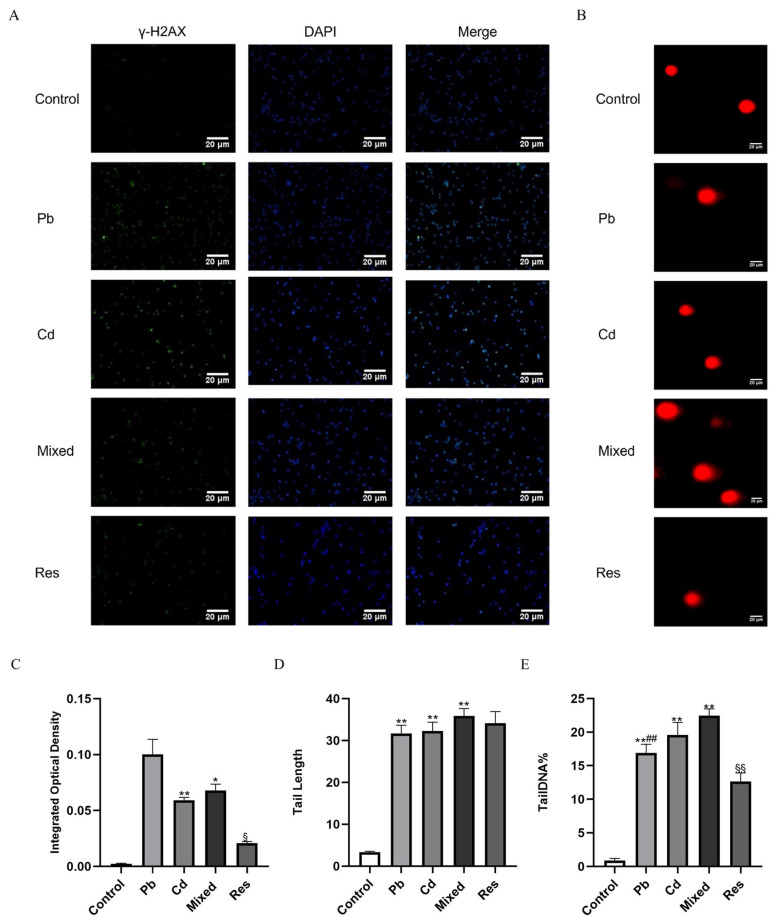
DNA damage markers induced by Pb and/or Cd in TK6 cells. (**A**,**C**) representative immunofluorescence staining images and quantification of γ-H2AX fluorescence intensity in TK6 cells. (**B**,**D**,**E**) representative the Comet assay images and quantitative analyses of Tail Length and Tail DNA%. Note: * *p* < 0.05, ** *p* < 0.01 for Pb and/or Cd group vs. the control group; while ^##^ *p* < 0.01 for the single exposure groups vs. the mixed group; ^§^ *p* < 0.05, ^§§^ *p* < 0.01 for the resveratrol group vs. the mixed group.

An immunofluorescence staining assay was used to assess the DNA damage marker γ-H2AX levels (Figure 3A,C). γ-H2AX fluorescence intensity was significantly higher in the Pb or Cd individual group than the control group (*p* < 0.05), and was further increased in the Pb and Cd mixed group. In contrast, the resveratrol intervention group significantly reduced γ-H2AX fluorescence compared with the mixed group (*p* < 0.05; Figure 3A,C). Similarly, the Comet assay results showed minimal DNA damage in the control group, which exhibited compact comet heads without obvious tails (Tail length, 3.35 ± 0.21 μm; Figure 3B,D). In Pb or Cd individual group, Tail Length (Pb: 31.66 ± 2.00 μm; Cd: 32.24 ± 2.14 μm) and Tail DNA% were markedly increased (Figure 3D,E), and these changes were further exacerbated in the Pb + Cd mixed exposure group (Tail Length: 35.88 ± 1.76 μm; Tail DNA%: 22.47 ± 1.01%; Figure 3D,E). Resveratrol treatment group markedly attenuated the DNA damage induced by Pb + Cd co-exposure group, as evidenced by significantly reduced γ-H2AX levels and Tail DNA% (*p* < 0.05; Figure 3C,E). Furthermore, two-way ANOVA revealed an antagonistic interaction between Pb and Cd on γ-H2AX fluorescence intensity, Tail DNA% and Tail Length (*F*_γ-H2AX = 35.819, *p* < 0.001; *F*_Tail DNA% = 27.096, *p* < 0.001; *F*_Tail length = 51.246, *p* < 0.001; Figure S1).

### 3.4. Pb and Cd Caused Cell Cycle Arrest and Apoptosis in TK6 Cells

**Figure 4:**
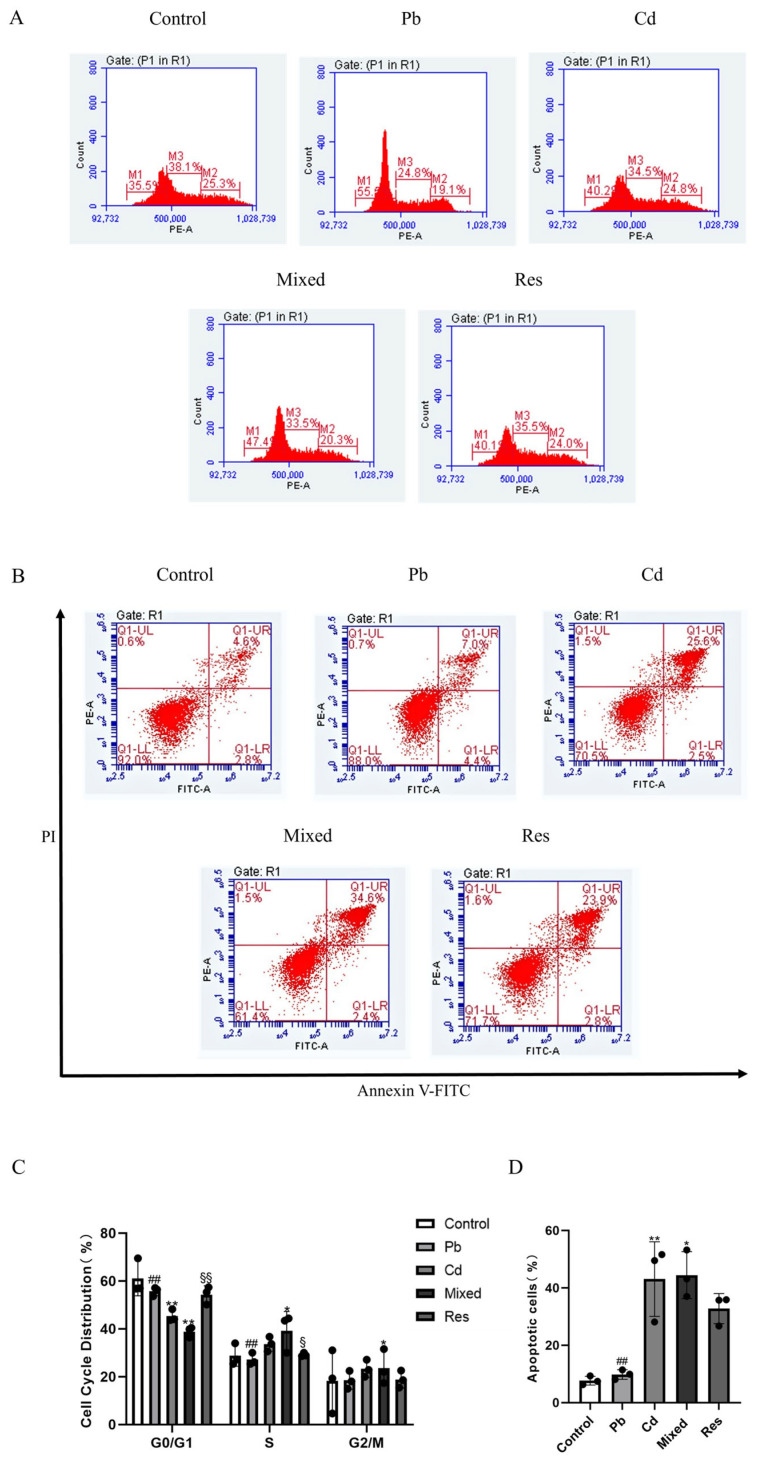
Cell cycle distribution and apoptosis rate of TK6 cells induced by Pb and/or Cd. (**A**,**C**) representative flow cytometry histogram of cell cycle distribution and quantification of S-phase. (**B**,**D**) representative flow cytometry plots of apoptosis detected by Annexin V-FITC/PI assay and quantification of apoptotic rate. Note: * *p* < 0.05, ** *p* < 0.01 for Pb and/or Cd group vs. the control group; while ^##^ *p* < 0.01 for single exposure groups vs. the mixed group; ^§^ *p* < 0.05, ^§§^ *p* < 0.01 for the resveratrol group vs. the mixed group.

Compared with the control group, exposure to Pb or Cd alone, as well as in combination, significantly increased the proportion of S-phase cells and decreased the proportion of G2/M-phase cells (*p* < 0.05; Figure 4A,C). Compared with the mixed group, the single-exposure group and the resveratrol group showed a lower proportion of S-phase and G0/G1-phase cells and a higher proportion of G2/M-phase cells (*p* < 0.05; Figure 4A,C). Apoptosis was quantified by flow cytometry in each group (Figure 4B,D). The late apoptosis rate in the lead-only group and the cadmium-only group was higher than that in the control group, indicating that both lead and cadmium can induce apoptosis, while the apoptosis rate in the resveratrol group (32.77 ± 3.03%) was lower than that in the mixed exposure group (Figure 4B,D). Two-way ANOVA further showed that co-exposure to Pb and Cd exerted an antagonistic interaction on the proportion of S-phase and G2/M-phase (*F*_S = 69.794, *p* < 0.01; *F*_G2/M = 85.763, *p* < 0.01; Figure S1).

### 3.5. Pb and Cd Inhibited DNA Repair Genes and Promoted Apoptosis-Related Gene Expression in TK6 Cells

**Figure 5:**
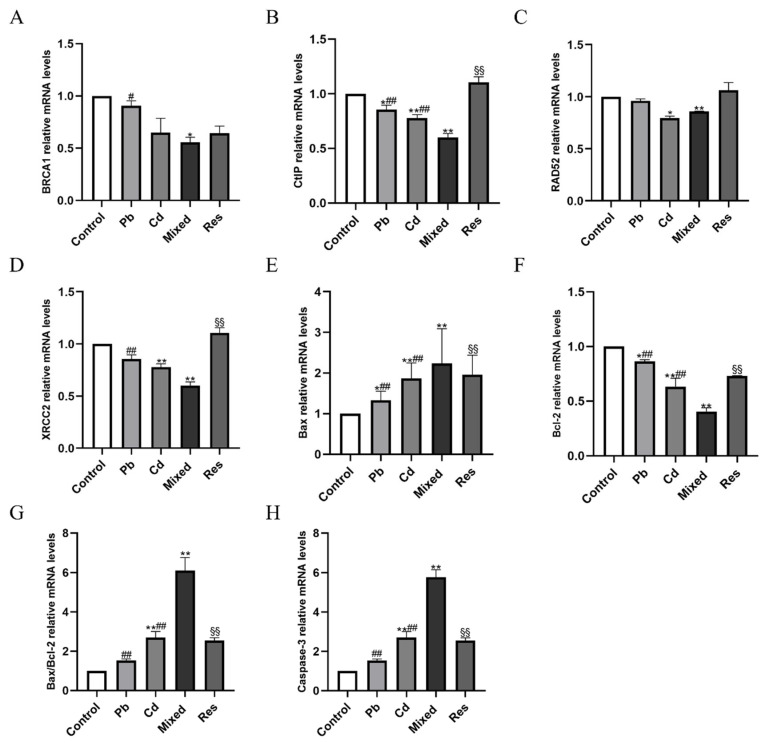
Relative mRNA expressions of DNA repair genes and apoptosis pathway after exposure to Pb and/or Cd in TK6 cells. (**A**–**D**) showed the relative mRNA expressions of *BRCA1*, *CtIP*, *RAD52* and *XRCC2*; (**E**–**H**) showed the relative mRNA expressions of *Bax*, *Bcl-2*, *Bax/Bcl-2* and *Caspase-3*. Note: * *p* < 0.05, ** *p* < 0.01 for Pb and/or Cd group vs. the control group; while ^#^ *p* < 0.05, ^##^ *p* < 0.01 for single exposure groups vs. the mixed group; ^§§^ *p* < 0.01 for the resveratrol group vs. the mixed group.

Compared with the control group, mRNA expression levels of DNA repair genes, including *BRCA1*, *CtIP*, *RAD52*, and *XRCC2,* were downregulated in the Pb or Cd group (*p* < 0.05; Figure 5A–D). In contrast, relative to the Pb + Cd mixed exposure group, *BRCA1*, *CtIP*, and *XRCC2* expression levels were significantly increased in both the single-exposure and resveratrol group (*p* < 0.05). *RAD52* was upregulated in the Pb-only group and resveratrol group (*p* < 0.05) but remained decreased in the Cd group (*p* < 0.05; Figure 5A–D). These findings suggest that Pb and Cd exposure suppressed DNA repair gene expression, with resveratrol partially reversing these changes. As for apoptosis-related genes, the expressions of *Bax*, *Caspase-3*, and the *Bax/Bcl-2* ratio were significantly increased in the single-exposure groups, whereas *Bcl-2* expression was decreased compared with the control group (*p* < 0.05; Figure 5E–H). Compared with the Pb + Cd mixed group, *Bax*, *Caspase-3*, and the *Bax/Bcl-2* ratio were reduced in both the single-exposure and resveratrol group, while *Bcl-2* expression was significantly upregulated (*p* < 0.05; Figure 5E–H). Two-way ANOVA further revealed an antagonistic interaction between Pb and Cd on the mRNA expressions of DNA repair genes (*BRCA1*, *CtIP*, *RAD52*, *XRCC2*), and apoptosis-related genes (*Bax*, *Bcl-2* and *Caspase-3*) (*F_BRCA1* = 26.822, *p* < 0.01; *F_CtIP* = 14.840, *p* < 0.01; *F_RAD52* = 28.763, *p* < 0.01; *F_XRCC2* = 22.132, *p* < 0.01; *F_Bax* = 50.056, *p* < 0.01; *F_Bcl-2* = 55.622, *p* < 0.01; *F_Bax/Bcl-2* = 19.638, *p* < 0.01, *F_Caspase-3* = 25.939, *p* < 0.01; Figure S2).

### 3.6. Pb and Cd Downregulated DNA Repair Proteins and Upregulated Apoptosis-Related Proteins in TK6 Cells

**Figure 6:**
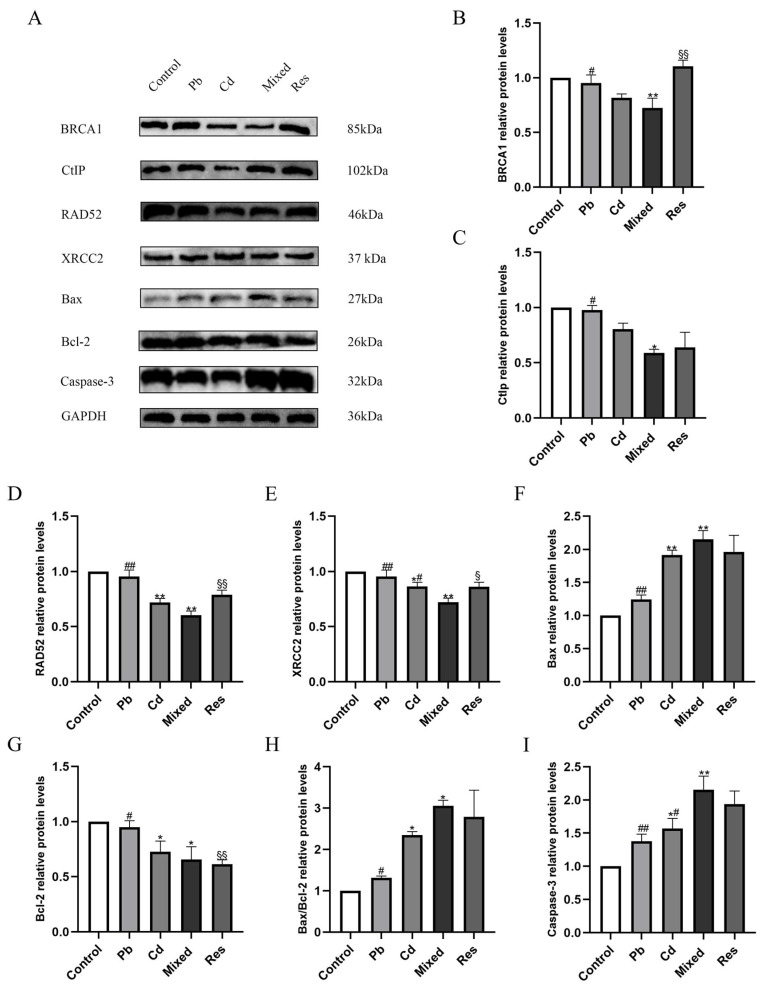
Relative protein expressions of DNA repair and apoptosis pathway after exposure to Pb and/or Cd in TK6 cells. (**A**–**E**) representative Western blotting images and quantitative protein levels of BRCA1, CtIP, RAD52 and XRCC2; (**A**,**F**–**I**) representative Western blotting images and quantitative protein levels of Bax, Bcl-2, Bax/Bcl-2 and Caspase-3. Note: * *p* < 0.05, ** *p* < 0.01 for Pb and/or Cd group vs. the control group; while ^#^ *p* < 0.05, ^##^ *p* < 0.01 for the single exposure group vs. the mixed group; ^§^ *p* < 0.05, ^§§^ *p* < 0.01 for the resveratrol group vs. the mixed group.

For DNA repair proteins, single exposure to lead and Cd downregulated BRCA1 and RAD52 expressions (*p* < 0.05; Figure 6A,B). Compared with the Pb + Cd mixed group, the BRCA1 and RAD52 protein levels were increased in the single metal exposure or resveratrol group (Figure 6A,B). Single metals also downregulated CtIP and XRCC2 (Figure 6A,C–E). Compared with the mixed group, CtIP and XRCC2 were upregulated in the single exposure group and the resveratrol group (Figure 6A,C–E). With respect to apoptosis-related proteins, single exposure to Pb or Cd increased Bax expression and the Bax/Bcl-2 ratio, mildly downregulated Bcl-2, and increased Caspase-3 expression compared to the control group. In contrast, both single-metal and resveratrol markedly reduced Bax, Caspase-3, and the Bax/Bcl-2 ratio compared to the mixed group (Figure 6A,F–I). Two-way ANOVA revealed an antagonistic interactions on Bcl-2, BRCA1, and the Bax/Bcl-2 ratio (*F*_Bcl-2 = 5.739, *p* = 0.043; *F*_BRCA1 = 6.768, *p* = 0.032; *F*_Bax/Bcl-2 = 5.739, *p* = 0.043; Figure S3).

## 4. Discussion

Pb and Cd are widely present in lead-smelting operations, battery manufacturing, and electroplating. These heavy metals can enter the human body via ingestion, inhalation, or dermal contact and accumulate over time, leading to systemic toxicity. Previous studies have shown that exposure to Pb and Cd alone induces in vitro cytotoxicity in a clear dose–response manner [31]. Consistent with our findings, various toxicants, including hydroquinone and Pb, have been reported to reduce TK6 cell viability in a dose-dependent manner. Although many investigations have focused on Pb or Cd individual exposure, Pb and Cd often co-occur in the environment, and their combined effects may be additive, synergistic, or antagonistic [32].

Several studies have suggested that co-exposure to Pb and Cd can be more cytotoxic than single-metal exposure, frequently via synergistic interactions [33,34,35]. Our literature review revealed that blood lead levels among workers in the lead-acid battery industry generally range from 400 to 750 μg/L, and can exceed 1000 μg/L in cases of severe exposure [36,37]. Blood cadmium levels in populations with occupational cadmium exposure usually range from 3 to 30 μg/L, and urine cadmium can be as high as 27.6 μg/g creatinine [38,39]. Considering that occupational exposure is chronic and long-term, whereas in vitro experiments reflect short-term acute exposure, and acknowledging that blood concentrations in in vitro studies are typically tens to hundreds of times higher than those seen in occupational settings, the experimental doses in this study were determined based on the IC_50_ values obtained from exposing TK6 cells to lead and cadmium. Building on this, we examined Pb and/or Cd toxicity in TK6 cells to clarify potential interaction patterns and underlying mechanisms. We observed cell-cycle arrest and apoptosis after Pb + Cd co-exposure, accompanied by oxidative stress and DNA damage, together with interference with the DNA repair pathway. Notably, two-way ANOVA-based interaction analyses indicated endpoint-specific, non-additive behavior, with evidence of antagonism for several readouts, even though overall injury remained greater than in the control group.

Disruption of intracellular redox homeostasis by heavy metals can perturb nucleotide integrity and drive oxidative DNA damage and genomic alterations [40]. Oxidative stress is characterized by excessive generation of ROS and impairment of antioxidant defenses [41]. Previous studies have reported positive associations between urinary Pb levels and oxidative-stress biomarkers, and both Pb and Cd can generate ROS (e.g., superoxide) and interact with antioxidant enzymes [42,43,44]. Previous studies have shown a strong correlation between the levels of lead and cadmium in the blood and biochemical indicators of oxidative stress [45,46]. In our study, co-exposure to lead and cadmium significantly elevated oxidative stress in TK6 cells, which is consistent with previous research [29]. Specifically, this effect was reflected by elevated ROS levels; similarly, exposure to 10 μM Pb combined with 2.5 μM Cd significantly upregulated ROS in PC12 cells [31], further supporting our results. In addition, GSSG levels increased while GSH content decreased. In contrast, exposure to 1.5 μM Pb and 1 μM Cd slightly increased GSH levels in HepG2 cells [47], possibly due to differences in experimental dose or cell type. Furthermore, co-exposure to lead and cadmium significantly increased MDA content while decreasing CAT and SOD levels. Previous studies have similarly shown that osteoblasts exposed to 1 and 10 μM Cd or Pb exhibited elevated MDA content and reduced CAT and SOD activity [48], consistent with our findings. These results are in line with in vivo experiments showing that co-exposed rats exhibit increased MDA and decreased SOD, CAT, and GPx [49]. Collectively, the suppression of GSH and SOD supports an oxidative-stress mechanism arising from Pb–Cd interplay.

Multiple studies have demonstrated that Pb and Cd induced apoptosis, closely linked to DNA damage [6,14,16,17]. Elevated γ-H2AX levels and Tail DNA% in Comet assay are sensitive markers of genotoxicity [50]. A substantial body of literature has reported significant associations between Pb or Cd exposure and DNA damage [2,7,51], and time- and dose-dependent manners between Pb and Cd have been observed in both mammalian and plant models [9,52]. In agreement with these findings, we observed increased γ-H2AX levels, Tail Length, and Tail DNA% in the Pb + Cd combined group compared with the single-exposure group. Interestingly, two-way ANOVA indicated antagonistic interactions for these DNA damage endpoints. Similar context-dependent interactions have been reported previously: adding Pb to low Cd concentrations increased DNA damage, whereas adding Pb to high Cd concentrations decreased it, consistent with competitive binding at the cell surface that modulates metal uptake and toxicity [53].

DNA damage activates checkpoint signaling that promotes cell-cycle arrest and, if damage persists, apoptosis to preserve genomic stability [54]. Pb or Cd individual exposure can induce S- or G2-phase arrest [55,56]. In our study, Pb and Cd co-exposure induced apoptosis, with downstream changes in Bcl-2 family members, namely, elevated Bax protein expression and decreased Bcl-2 protein level, and an increased Bax/Bcl-2 protein ratio. One plausible explanation is that Pb may compete with Cd for calcium channels or other transport pathways. This mechanistic hypothesis warrants targeted validation.

The DNA repair system is essential for counteracting metal-induced genomic instability [57,58]. Key DNA repair pathways include homologous recombination (HR), non-homologous end joining (NHEJ), nucleotide excision repair (NER), and base excision repair (BER) [59]. Pb has been reported to inhibit DNA-PK activity, thereby impairing NHEJ and secondarily engaging RAD51-dependent HR, whereas Cd can compromise NER by inhibiting critical enzymes [55,60]. In our study, several DNA repair proteins were downregulated in the combined exposure group, notably BRCA1 and representative HR components, prompting us to focus on HR-mediated repair. We found that Pb and Cd co-exposure disrupted RAD52, XRCC2, and CtIP expressions, which would exacerbate the burden of DNA double-strand breaks. HR provides high-fidelity repair of DSBs and is often recruited under severe DNA damage [61]. We propose that Pb and Cd exposure induce oxidative stress and alter transcription factor activity, thereby reducing DNA repair gene expression and limiting repair capacity; however, this conjecture needs further verification. Resveratrol, as a natural polyphenolic antioxidant, enhances the efficiency of NER and mismatch repair pathways, or activates repair proteins such as BRCA1 and Rad51, promoting HR repair, thereby improving the ability to repair double-strand breaks [62,63,64,65]. This study observed that Pb and Cd co-exposure exhibited a significant antagonistic effect on the suppression of the DNA repair system, suggesting a potential intervention target against heavy metal genotoxicity.

This study has several limitations. First, we used human lymphoblastoid TK6 cells as an in vitro model; findings from a single cell type may not be generalized to other tissues or organisms. Further animal studies are needed to validate our results. Second, although we detected mRNA and protein expression changes in the DNA repair and apoptosis pathway, more work is required to delineate upstream regulatory mechanisms, including epigenetic modulation and the roles of metal transporters and channels. Despite these limitations, our work systematically characterizes the effects of Pb and Cd co-exposure on oxidative stress, DNA damage, cell cycle arrest, DNA repair and apoptosis pathway, providing mechanistic insights for the prevention and treatment of heavy metal combined toxicity.

## 5. Conclusions

The current study demonstrated that combined exposure to Pb and Cd induces oxidative stress in TK6 cells and leads to significant genotoxicity. This process was accompanied by dysregulation of DNA repair-related protein expression, ultimately triggering cell cycle arrest and apoptosis. Notably, these two metals exhibit an antagonistic interaction in terms of oxidative damage, cell cycle progression, and apoptosis. Furthermore, resveratrol demonstrated a partial protective effect by modulating ROS levels, SOD activity, γ-H2AX expression, Tail DNA%, and the expression of proteins involved in DNA repair and apoptotic pathways.

## Figures and Tables

**Table 1:** Primer sequences for real-time quantitative PCR.

No.	Gene Name	Accession Number	Sequence (5′ to 3′)	Products (bp)
1	*BRCA1*	NM_007297	F: GAAACCGTGCCAAAAGACTTC	88
			R: CCAAGGTTAGAGAGTTGGACAC	
2	*CtIP*	NM_002894	F: CAGGAACGAATCTTAGATGCACA	123
			R: GCCTGCTCTTAACCGATCTTCT	
3	*RAD52*	NM_134424	F: CCAGAAGGTGTGCTACATTGAG	145
			R: ACAGACTCCCACGTAGAACTTG	
4	*XRCC2*	NM_005431	F: TGCTTTATCACCTAACAGCACG	124
			R: TGCTCAAGAATTGTAACTAGCCG	
5	*Bax*	NM_138763	F: CCCGAGAGGTCTTTTTCCGAG	155
			R: CCAGCCCATGATGGTTCTGAT	
6	*Bcl-2*	NM_000657	F: GGTGGGGTCATGTGTGTGG	89
			R: CGGTTCAGGTACTCAGTCATCC	
7	*Caspase-3*	NM_004346	F: CATGGAAGCGAATCAATGGACT	139
			R: CTGTACCAGACCGAGATGTCA	
8	*GAPDH*	NM_001256799	F: GGAGCGAGATCCCTCCAAAAT	197
			R: GGCTGTTGTCATACTTCTCATGG	

## Data Availability

The original contributions presented in this study are included in the article/Supplementary Materials. Further inquiries can be directed to the corresponding author.

## Supplementary Materials

The following supporting information can be downloaded at: https://www.mdpi.com/article/10.3390/toxics14040341/s1, Figure S1. Interaction plots of oxidative stress indicators, genetic damage markers, cell cycle and apoptosis after exposure to Pb and Cd in TK6 cells; Figure S2. Interaction plots of relative mRNA expressions of DNA repair genes and apoptosis pathway after exposure to Pb and Cd in TK6 cells; Figure S3. Interaction plots of relative protein expressions of DNA repair genes and apoptosis pathway after exposure to Pb and Cd in TK6 cells.

## Abbreviations

The following abbreviations are used in this manuscript:

Pb	Lead
Cd	Cadmium
IARC	International Agency for Research on Cancer
Res	Resveratrol
ROS	Reactive Oxygen Species
GSH	Glutathione
GSSG	Oxidized Glutathione
CAT	Catalase
MDA	Malondialdehyde
SOD	Superoxide Dismutase
RT-qPCR	Real-time quantitative PCR
