# Supplementary material for: Oxidative Stress, DNA Damage, DNA Repair Inhibition, and Apoptosis Induced by Lead and Cadmium Combined Exposure in TK6 Cells
Source: Toxics. 2026 Apr 18;14(4):341. doi: 10.3390/toxics14040341 (PMC13120129; doi:10.3390/toxics14040341)
Supplement: Supplementary file 1 [file toxics-14-00341-s001.zip › toxics-4165117-supplementary.pdf]

Supplementary Materials

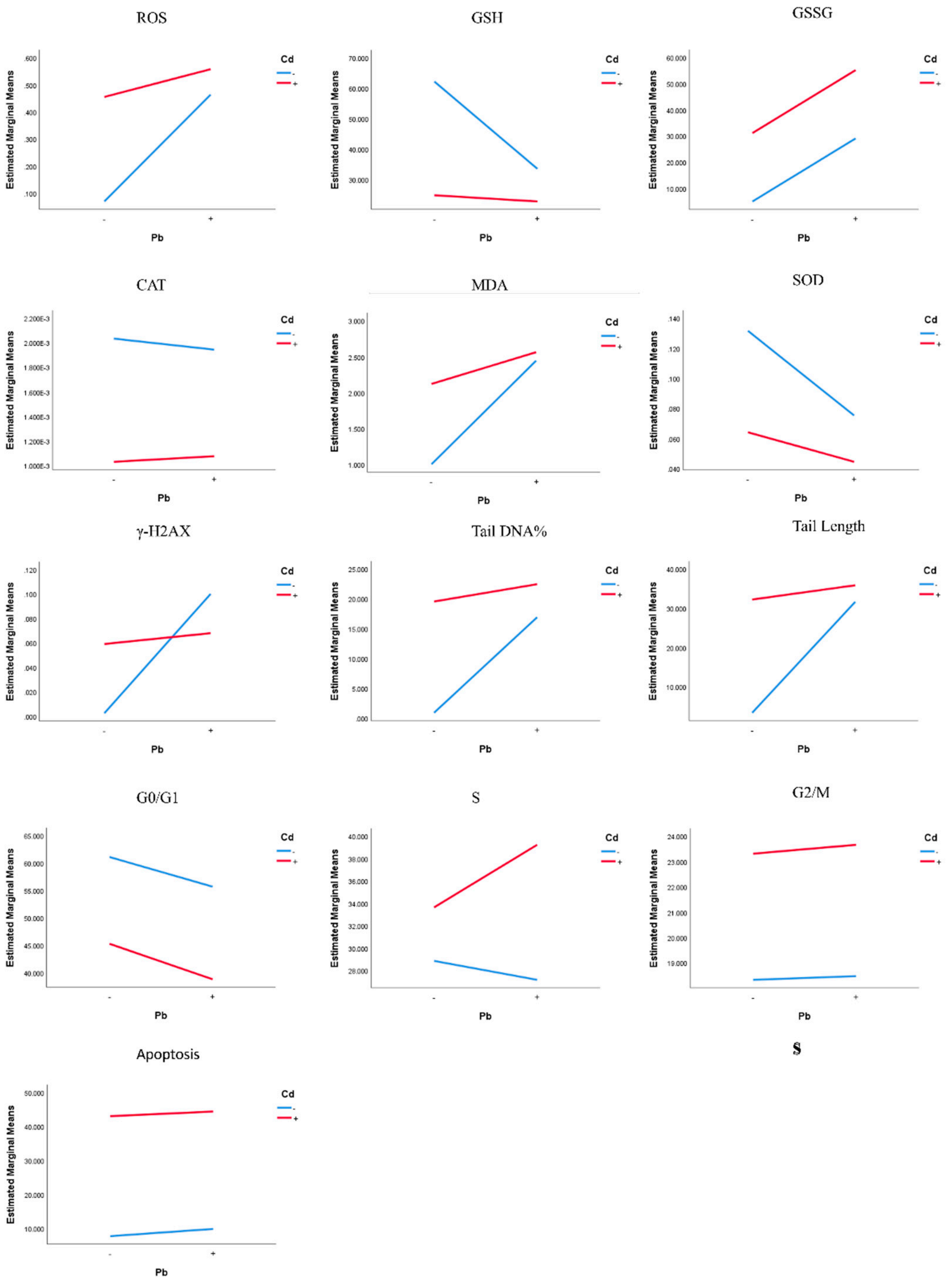

Figure S1 Interaction plots of oxidative stress indicators, genetic damage markers, cell cycle and apoptosis after exposure to Pb and Cd in TK6 cells

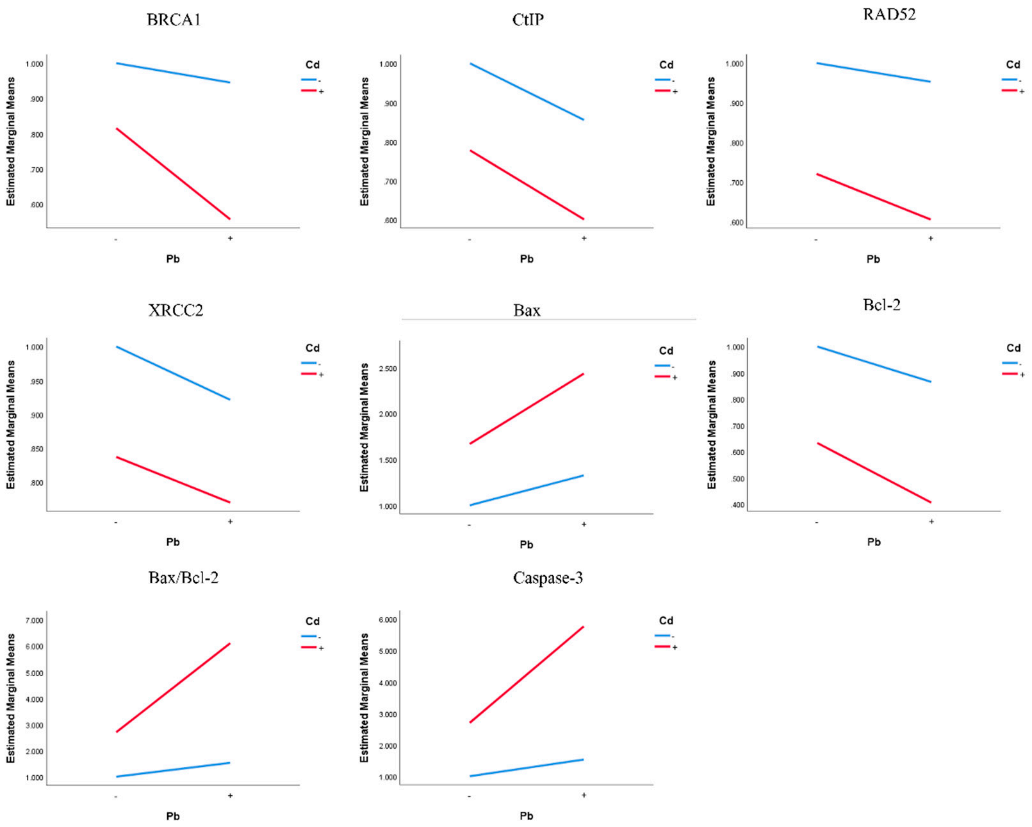

Figure S2 Interaction plots of relative mRNA expressions of DNA repair genes and apoptosis pathway after exposure to Pb and Cd in TK6 cells

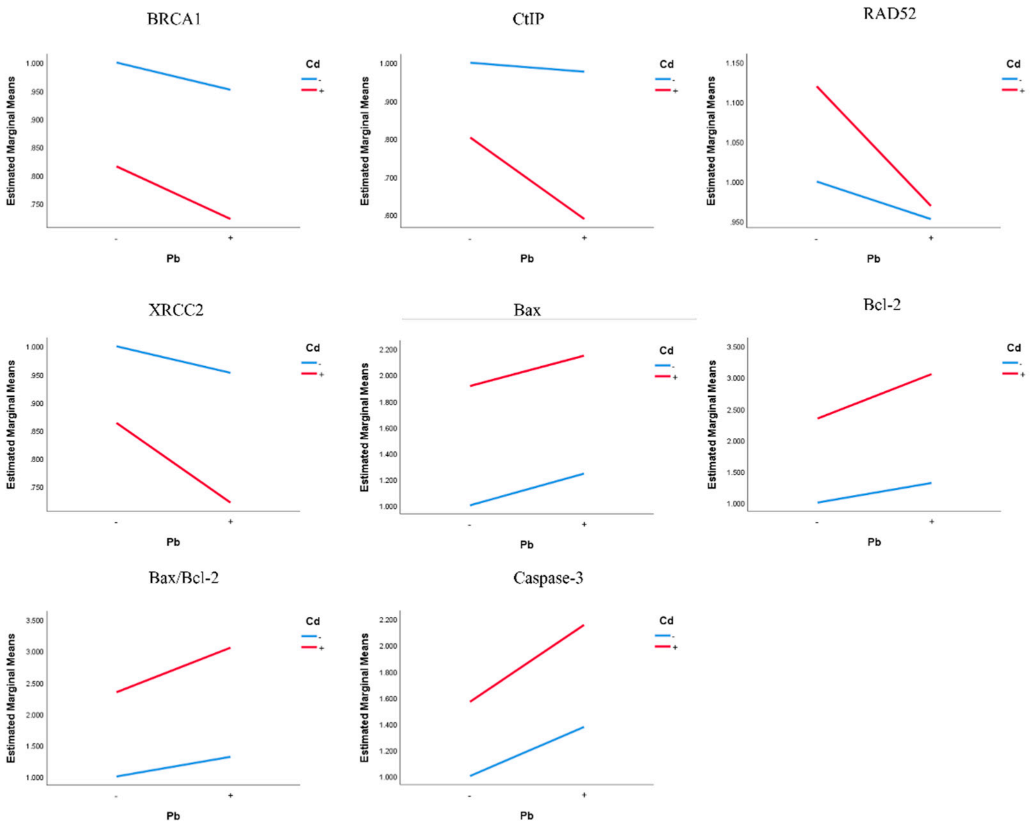

Figure S3 Interaction plots of relative protein expressions of DNA repair genes and apoptosis pathway after exposure to Pb and Cd in TK6 cells.
